# Radiobiology studies of gold nanoparticle‐enhanced radiosensitization in proton therapy

**DOI:** 10.1002/mp.70573

**Published:** 2026-07-21

**Authors:** N. Fuster‐Martínez, D. H. Saucedo‐Cuberes, M. C. Jiménez‐Ramos, D. Esperante, J. M. Espino, J. Fuster, J. F. Belén‐Aguilar, L. González‐López, P. Huertas, S. Jimeno, I. Mingarro, D. Pascual, D. Suárez‐García, M. J. García‐Murria

**Affiliations:** ^1^ Instituto de Física Corpuscular (CSIC‐UV) Paterna Spain; ^2^ Centro Nacional de Aceleradores, CNA Sevilla Spain; ^3^ Departamento de Física Aplicada II Universidad de Sevilla Sevilla Spain; ^4^ Departamento de Física Atómica, Molecular y Nuclear Universidad de Sevilla Sevilla Spain; ^5^ Facultat Ciències Biològiques, Institut Universitari de Biotecnologia i Biomedicina (BIOTECMED) Universitat de València Burjassot Spain; ^6^ Facultad de Biología Universidad de Sevilla Sevilla Spain; ^7^ Centro Andaluz de Biología Molecular y Medicina Regenerativa‐CABIMER Universidad de Sevilla‐CSIC‐Universidad Pablo de Olavide Sevilla Spain

**Keywords:** gold nanoparticles, hadrontherapy, nanomedicine, radiobiology, radiosensitization

## Abstract

**Background:**

Approximately 50% of newly diagnosed cancer patients worldwide receive radiotherapy, most commonly with X‐rays. Proton therapy has emerged as a promising alternative, offering complementary advantages over conventional X‐rays due to its highly localized energy deposition at the Bragg peak, thereby reducing radiation exposure to surrounding healthy tissues. In recent years, advances in both technology and biomedicine have driven significant progress in proton therapy, with ongoing research aiming to minimize toxicity and long‐term side effects while improving patients' quality of life. An emerging research direction investigates the combination of proton therapy with irradiated nanoparticles acting as radiosensitizers. This approach seeks synergistic effects that could further improve therapeutic outcomes. These effects arise from a complex interplay of physical, chemical, and biological mechanisms. Although previous in vitro and in vivo studies report promising radiosensitization effects of nanoparticles, the literature also includes contradictory results, and the underlying mechanisms remain under debate. This highlighting the need for systematic studies under well‐controlled irradiation conditions of different nanoparticles and beam parameters, which are often difficult to perform due to the limited availability of particle accelerators and facilities suitably adapted for such experiments.

**Purpose:**

This work investigates the radiosensitizing effect of gold nanoparticles under proton irradiation and optimizes the irradiation setup to enable more efficient and reproducible future experiments.

**Methods:**

HeLa cells were used as the biological model for the radiobiological experiments. Irradiation conditions were optimized by incorporating a Mylar film into the culture plates to maintain a minimal medium volume and ensure rapid post‐irradiation coverage. Cells with and without AuNPs were irradiated with 12.6 MeV proton beams. Radiosensitivity was assessed using clonogenic survival assays, and the radiosensitizing effect, together with its associated uncertainty, was quantified. DNA damage analysis was also performed to corroborate the observed effects and explore underlying mechanisms.

**Results:**

The radiobiology experiments confirmed and quantified a significant radiosensitizing effect of gold nanoparticles (AuNPs) on HeLa cells at doses from 4 Gy, with Sensitization Enhancement Ratio (SER) values of 1.53 ± 0.07 in 2022 and 1.30 ± 0.20 in 2023 using 50 nm diameter AuNPs. Experiments in 2023 with 20 nm diameter AuNPs yielded a comparable SER of 1.38 ± 0.18, indicating no significant size‐dependent differences. The radiosensitizing effect exhibited dose‐dependent amplification, with higher doses producing greater cell killing. Linear‐Quadratic (LQ) model fits showed an increased α parameter in the presence of AuNPs, consistent with an increase in non‐repairable damage. This trend was further supported by double‐strand break assays. Furthermore, oxidative stress assays showed that AuNPs do not increase basal ROS levels, but enhance ROS production under oxidative stress conditions.

**Conclusion:**

The radiobiology experiments presented quantitatively confirm that both 20 and 50 nm diameter AuNPs enhance the radiosensitivity of HeLa cells under proton irradiation. The radiosensitizing effect increases with dose, indicating a dose‐dependent response, while no significant differences were observed between nanoparticle sizes. Consistent trends of increased DNA double‐strand breaks further support the observed radiosensitization. Evidence from oxidative stress assays suggests that AuNPs may enhance ROS production under oxidative conditions, potentially contributing to the observed radiosensitization and DNA damage.

## 1 INTRODUCTION

Radiotherapy is a key cancer treatment, with about 50% ^1^ of newly diagnosed patients requiring it. As the population grows and ages, demand is expected to rise. X‐rays are most commonly used, but proton therapy has shown significant and complementary advantages over X‐rays, due to the very localized energy deposition on the Bragg peak, minimizing the dose to normal tissue.^2^ Proton therapy has advanced significantly over the past two decades and as it evolves, new technologies are being explored to reduce toxicity and improve patient quality of life. Current radiotherapy approaches still face limitations in treating advanced, radioresistant, and hypoxic tumors. One promising strategy involves incorporating radiosensitizers directly into the tumor to enhance the effectiveness of radiotherapy.^3^, ^4^ This approach has the potential to improve the sensitivity of the tumor to radiation, resulting in more targeted radiation‐induced damage and potentially better therapeutic outcomes.

High‐Z metallic nanoparticles (NPs) have been widely investigated as radiosensitizers with different radiotherapy modalities showing promising results both, in in vitro and in vivo experiments.^5^, ^6^ Specially, gold nanoparticles (AuNPs) have emerged as key candidates because of their excellent biocompatibility, ease of synthesis across a broad range of sizes, and the relative simplicity of surface functionalization through ligand attachment for targeted cancer cell delivery. Additionally, the enhanced permeability and retention (EPR) effect,^7^, ^8^, ^9^ resulting from leaky tumour vasculature and poor lymphatic drainage, facilitates their preferential accumulation in tumour tissues.

Numerous experimental and simulation studies with X‐rays have helped to elucidate the potential mechanisms involved^10^, ^11^ and currently, non‐functionalized hafnium oxide nanoparticles are undergoing clinical trials in the U.S., showing promising results.^12^, ^13^ Although the radiosensitization effect has been observed with protons, particularly using AuNPs,^14^, ^15^, ^16^, ^17^, ^18^, ^19^, ^20^, ^21^, ^22^, ^23^, ^24^, ^25^ the number of studies is limited, and some results remain controversial, showing effects greater than predicted by simulations.^26^, ^27^, ^28^, ^29^, ^30^, ^31^ In addition, differences in nanoparticle properties, incubation times, and beam characteristics complicate direct comparisons across experimental studies.

Monte Carlo‐based simulation studies suggest that the primary physical mechanism underlying proton‐induced radiation enhancement by AuNPs is the increased production of low‐energy electrons (LEE), which deposit their energy within the nanoscale vicinity of the nanoparticles. These electrons arise predominantly from ionization processes, Auger cascades, and the emission of fluorescent X‐rays. While significant progress has been made in modeling radiation–nanoparticle interactions, the contribution of the physical stage to the overall biological response remains incompletely understood. At the nanoscale, simulations predict enhanced secondary electron production and localized dose deposition around individual nanoparticles; however, due to the very short range of these electrons, such effects are strongly reduced when extended to realistic geometries and irradiation conditions. This leads to a persistent discrepancy between predicted physical dose enhancement and experimentally observed biological outcomes. Consequently, experimental investigations that systematically explore the influence of physically relevant parameters, such as beam characteristics and nanoparticle properties, are essential to provide phenomenological insight as well as experimental data for validating and constraining future developments in physics‐based and physicochemical models, which are central tools in medical physics and an active area of research. Moreover, the implementation of such studies in proton beams represents a significant experimental challenge, requiring dedicated instrumentation and precise beam characterization. Advancing these experimental capabilities is therefore a key step for enabling reliable and reproducible research in this field. Subsequently, the LEEs can trigger the formation of reactive oxygen species (ROS), such as hydroxyl radicals and superoxide anions, thereby contributing to oxidative stress, mitochondrial dysfunction, and ultimately cell death. The enhancement of ROS production in the presence of AuNPs has been investigated both through Monte Carlo simulations^32^, ^33^ and radiobiological experiments.^34^, ^35^

However, discrepancies between computational predictions and experimental measurements indicate that the radiosensitizing effect is not yet fully understood and that current physical and chemical models may have intrinsic limitations and remain incomplete given the complexity of the problem. This raises also the possibility that additional, indirect biological mechanisms may contribute to the overall enhancement. For example, it has been proposed that AuNPs may interfere with the cell cycle by prolonging radiation‐sensitive phases, suppress antioxidant proteins such as thioredoxin reductase (thereby further increasing intracellular ROS levels), or impair DNA repair pathways.^12^ The potential contribution of direct DNA damage has also been examined using Monte Carlo simulations,^36^ but results consistently show that physical interactions alone generate insufficient DNA damage to account for the experimentally observed levels of radiosensitization.

Together, these findings highlight the need to identify and characterize complementary biological mechanisms of AuNP‐mediated radiosensitization beyond the well‐established pathway of radiation‐induced direct DNA damage.

Overall, while evidence supports the radiosensitizing potential of AuNPs through a complex interplay of physical, chemical, and biological mechanisms, further research is needed to clarify their exact role and optimize their clinical application^29^ as well as improving the mathematical models needed for their application in clinical treatment planning. This study aims to support these efforts through radiobiology experiments and the development of optimized experimental conditions and tools for future systematic studies with protons.

In this context, we investigated the radiosensitizing effect of AuNPs in HeLa cells exposed to 12.6 MeV proton irradiation, an energy within the Bragg peak region relevant for clinical proton therapy. Experiments were designed to setup and assess nanoparticle uptake, cytotoxicity, clonogenic survival, and DNA damage, aiming to clarify the physical and biological contributions of AuNPs to radiosensitization, as well as establishing optimized experimental conditions, including the development of a robotic irradiation system, to enable more efficient campaigns and reproducible future studies.

## 2 MATERIALS AND METHODS

### 2.1 Cell lines and growth conditions

Human epithelial cervical cancer cells (HeLa, ATCC® CCL‐2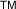) were cultured in Dulbecco's modified Eagle's medium (DMEM) (Gibco) supplemented with 10% fetal bovine serum (FBS) (Gibco), and penicillin‐streptomycin (P/S) (100 U/mL) (Gibco). All cells were grown at 37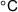, with 5% ${\mathrm{CO}}_{2}$.

### 2.2 AuNPs

Spherical AuNPs of 50 nm diameter from Cytodiagnostic with an optical density (netOD) of 10 have been purchased. The AuNPs are supplied in 0.1 mM phosphate buffered saline (PBS) and the concentration is of 3.51$\times 10^{11}$ particle per mL. In all cases, AuNPs were used at a concentration of 148 μg/mL, diluted in serum‐containing DMEM prior to cell treatment. 20 nm diameter AuNPs have also been purchased from the same company with similar properties except the concentration which corresponds to 6.54$\times 10^{11}$ particle per mL.

### 2.3 AuNP citotoxity assays

To study the cytotoxic effect of AuNPs, cells were seeded either in 96‐well plates (5000 cells/well) or in 24‐well plates (7$\times 10^{4}$ cells/well) and incubated with diluted AuNPs (148 μg/mL) for 1.5, 4, or 24 h. Cell viability was first assessed using the CellTiter‐Glo® Luminescent Cell Viability Assay (Promega), which quantifies ATP as an indicator of metabolically active cells. After washing, 100 μL of CellTiter‐Glo® reagent were added to each well, plates were gently shaken for 2 min and incubated for 10 min at room temperature in the dark. Luminescence was measured using a Victor X3 plate reader. In parallel, viability was also evaluated using the WST‐1 assay (Roche), which measures mitochondrial activity via tetrazolium salt reduction. After washing with PBS, 100 μL of medium containing 10 μL of WST‐1 reagent were added to each well. Plates were incubated for 1–4 h at 37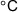 in the dark, and absorbance was measured at 450 nm using the Victor X3 reader. In both cases, viability was expressed as a percentage relative to the control after subtracting background values from blank wells (only cells no probe, NPs without cells). Apoptosis was analysed by flow cytometry using the FITC Annexin V Apoptosis Detection Kit (Immunostep Biotech), following the manufacturer's protocol. Cells were seeded in 24‐well plates (7$\times 10^{4}$ cells/well) and incubated with diluted AuNPs (148 μg/mL) for 2, 4, or 24 h. After each incubation period, cells were trypsinized for 5 min at 37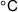, and the reaction was stopped with serum‐containing DMEM. Following centrifugation at 300 × g for 5 min and a single PBS wash, cells were resuspended in 100 μL of 1X binding buffer and stained with 5 μL of Annexin V‐FITC and 5 μL of propidium iodide (PI). Samples were incubated for 15 min at room temperature in the dark, followed by the addition of 400 μL of binding buffer and subsequent filtration. Flow cytometry was performed using a BD ${\mathrm{LSRFortessa}}^{\mathrm{TM}}$ cytometer, acquiring 10 000 events per sample. Data were analysed with ${\mathrm{FlowJo}}^{\mathrm{TM}}$ software, and cells were classified as viable (Annexin $V^{-}/PI^{-}$), early apoptotic (Annexin $V^{+}/PI^{-}$), late apoptotic or necrotic (Annexin $V^{+}/PI^{+}$), and necrotic (Annexin $V^{-}/PI^{+}$).

### 2.4 Cellular uptake measurements with AuNPs for Hela cells

Gold content in HeLa cells treated with AuNPs was quantified using inductively coupled plasma mass spectrometry (ICP‐MS) at the Atomic Spectroscopy Section of the Central Support Service for Experimental Research (SCSIE) of *Universitat de València*.^37^ HeLa cells were seeded in 12‐well plates and, on the following day, incubated with AuNPs in complete DMEM for 1.5, 4, 6, or 24 h. After incubation, the wells were washed twice with PBS to remove extracellular nanoparticles. Cells were trypsinized, collected by centrifugation, and resuspended in PBS. Samples were subsequently digested with acid for gold quantification. Digestion was performed using a high‐pressure microwave system (ETHOS EASY, Milestone) with a mixture of ultrapure nitric acid (${\mathrm{HNO}}_{3}$, 69%) and hydrochloric acid (HCl, 37%) (Scharlab, ppb‐trace analysis grade), reaching a maximum temperature of 220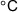. The resulting digests were diluted with ultrapure Milli‐Q water prior to analysis. Gold concentration was measured using an Agilent 7900 ICP‐MS system equipped with a Micromist concentric nebulizer, Scott‐type spray chamber, nickel interface cones, an off‐axis dual lens system, a hyperbolic quadrupole mass filter, and an octopole collision/reaction cell operating in helium mode to reduce polyatomic interferences. Quantification was performed using external calibration (0–1000 ppb) with iridium (Ir) as an internal standard. Certified reference standards of gold (1000 mg/L, High‐Purity Standards) and iridium (20 μg/mL, ISC Science) were used. The isotopes monitored were 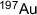 and 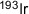. Each experimental condition was tested in triplicate. For each replicate, the mean and standard deviation of the gold concentration (μg/mL) were used to calculate the gold content per cell (ng Au/cell) and the number of nanoparticles per cell (NPs/cell), based on the manufacturer's specification that $3.5\times 10^{11}$ nanoparticles correspond to 445 μg of gold.

### 2.5 AuNPs localization

The internalization of AuNPs on the cells was studied by Transmission Electron Microscopy (TEM). HeLa cell cultures were grown in 8‐well chamber slides (μ‐Slide 8 Well, IBIDI) and incubated with AuNPs for 2, 4, or 24 h. Following incubation, cells were washed with PBS and fixed with 2.5% glutaraldehyde (pH 7.4) for 1 h at room temperature. After fixation, samples were washed with phosphate buffer (PB) and post‐fixed with 1% osmium tetroxide (${\mathrm{OsO}}_{4}$) for 1 hour. Dehydration was carried out through a graded ethanol series (30%–100%), and samples were subsequently embedded in LR‐White resin. Once the resin was polymerized, ultrathin sections (60–90 nm) were obtained using a Leica UC7 ultramicrotome and collected on copper grids. Prior to imaging, sections were contrasted with 2% uranyl acetate and Reynold's lead citrate solution. Samples were processed at the Sample Preparation Area of the Microscopy Section of SCSIE, *Universitat de València*. Imaging was performed using a Hitachi HT7800 transmission electron microscope operating at acceleration voltage of 100 kV. Digital images were acquired and analysed to evaluate the intracellular localization and distribution of the nanoparticles.

### 2.6 Effect of AuNPs in the production of intracellular ROS

HeLa cells were seeded in surface‐treated black 96‐well plates or 24‐well plates at a density of 20 000 cells/${\mathrm{cm}}^{2}$ and incubated overnight. After 24h, the culture medium was replaced with medium containing AuNPs (1:3 dilution), followed by a 24h incubation. For oxidative stress induction, tert‐butyl hydroperoxide (TBHP) (Thermo Fisher Scientific) was added at a dose corresponding to 500 μequivalents of oxidative capacity, and cells were incubated for 1h in PBS, in both AuNP‐treated and untreated conditions. Cells were then incubated with 11 μM of the ROS probe $CM-H_{2}\text{DCFDA}$ for 45 min at 37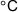. Parallel conditions using DMSO instead of the probe were included to correct baseline autofluorescence. After three PBS washes to remove $CM-H_{2}\text{DCFDA}$ in excess, fluorescence was measured using a Victor X3 plate reader (excitation: 490nm, emission: 530nm), with six independent wells per condition. Following fluorescence acquisition, cell viability was quantified via ATP measurement using CellTiter‐Glo® 2.0 reagent as described previously. Fluorescence signals were corrected by subtracting values from probe‐free controls, normalized to ATP levels (total of live cells), and further normalized to untreated cells in PBS (set as 100%) within each experiment. Data represent the mean of three independent biological replicates and statistical analysis was performed using one‐way ANOVA, comparing each treatment to the HeLa in PBS control. For qualitative analysis, HeLa cells from 24‐well plates were imaged using a Zeiss Axiovert 5 fluorescence microscope with 20x or 40x objectives. Images were acquired in transmitted light and green fluorescence channels and subsequently overlaid.

### 2.7 Proton irradiation setup

**FIGURE 1:**
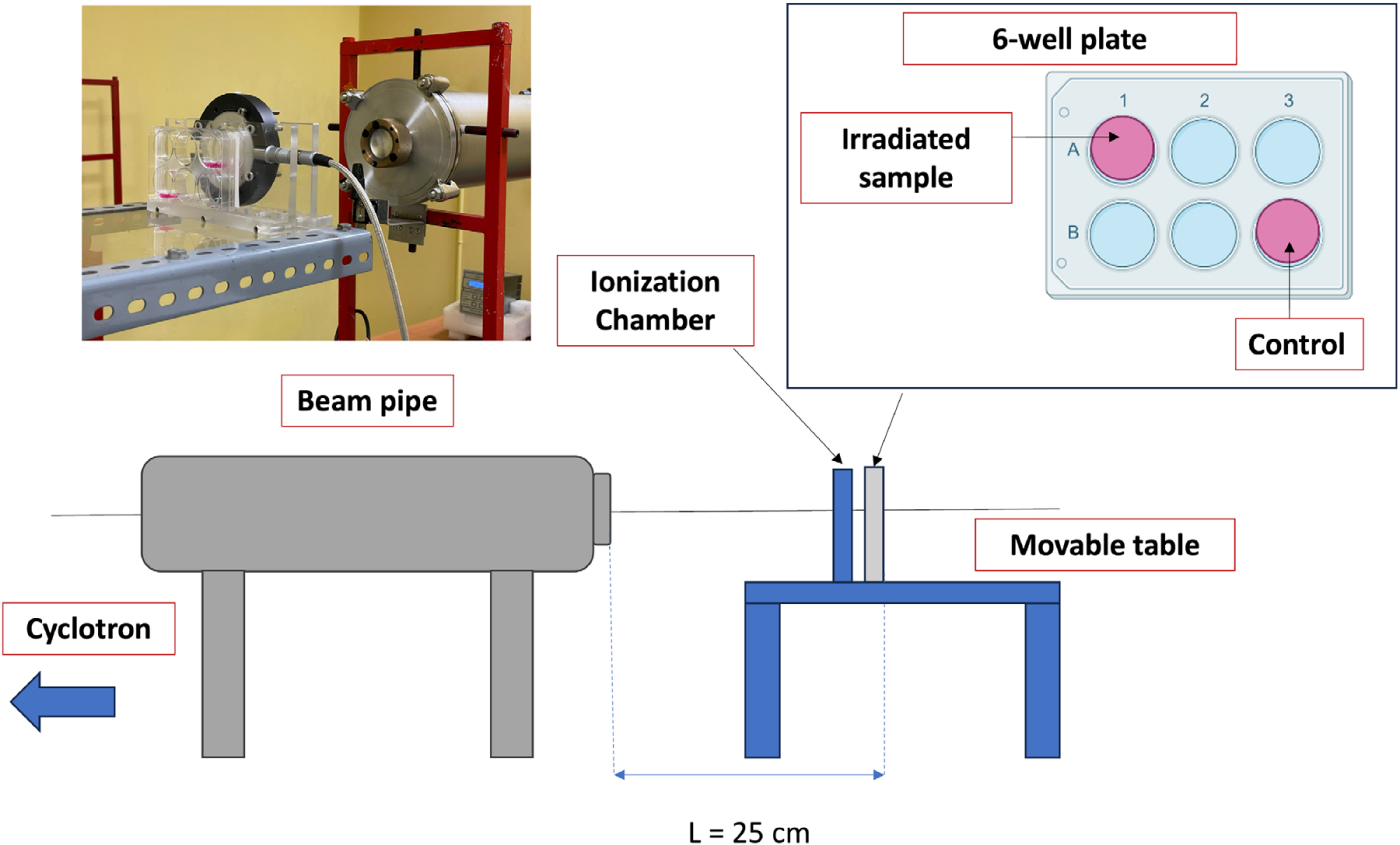
Experimental setup. Exit window of the CNA external beamline, followed by the ionization chamber used for dose measurements and a 6‐well plate positioned vertically with a 100 μm Mylar film, prepared for irradiation. The culture medium shown in the image is intentionally left to allow rapid coverage of the cells immediately after irradiation.

Proton irradiations were carried out at *Centro Nacional de Aceleradores* ^38^ (CNA, Universidad de Sevilla, CSIC, Junta de Andalucía, Seville, Spain) using the Cyclone 18/8 cyclotron (IBA, Ion Beam Applications, Louvain‐la‐Neuve, Belgium), which accelerates protons and deuterons up to 18 and 9 MeV, respectively. The cyclotron features eight target ports, seven of which are dedicated to the production of medical radioisotopes, while one port is used to transport the proton beam to an external beam line for interdisciplinary research. The experimental setup for radiobiological irradiations was optimized as described by Baratto‐Roldán et al.^39^. This setup, shown in Figure 1, allows the biological samples to be irradiated in a vertical orientation with a circular and homogeneous irradiation field with a diameter of 3.5 cm. The homogeneity of the irradiation field is verified prior to each experiment using Gafchromic EBT3 films. These films are irradiated up to 2‐4 Gy, subsequently scanned, and analysed with *ImageJ* software. These measurements are also used to confirm the correct alignment of the well‐plate centres with the proton beam axis. In order to reduce the time that the cells are without medium during the irradiation procedure, 500 μL of medium is left and the plate is covered with a 100 μm Mylar film. In this way, a more rapid coverage of the cells is achieved after irradiation.

Furthermore, an ionization chamber positioned upstream of the irradiation plate is used to perform in situ dose measurements during irradiation. The charge collected by the chamber is correlated with Monte Carlo simulation results to generate a calibration curve, which enables the estimation of the absorbed dose at the cell culture from the measured charge. The ionization chamber is mounted on a dedicated support structure designed to hold both the ionization chamber and the 6‐well plate.

The incorporation of the Mylar film into the irradiation setup required to update the dose calibration curve with respect to previous experiments reported by Baratto‐Roldán et al.^39^. Monte Carlo simulations were therefore performed to account for the presence of the film and to obtain an updated calibration curve for the modified configuration. These simulations were carried out using geant4 version 10.7.4 with the QGSP_BIC_HP_EMZ physics list, which is commonly employed for modelling proton interactions at low energies. The well was modelled as a cylindrical volume with a radius of 3.5 cm and a depth of 1.8 cm. A thin layer representing adherent cells was placed at the bottom, and simulations were performed assuming cell layer thicknesses of 20 and 40 μm to evaluate the influence of culture thickness on the average LET and deposited dose.

### 2.8 Clonogenic assays

All sample preparation and post‐irradiation processing were carried out at *Centro Andaluz de Biología Molecular y Medicina Regenerativa* ^40^ (CABIMER). HeLa cells were seeded exclusively in two extreme opposite plates from the 6‐well plates (as can be seen in Figure 1) 18 h prior to irradiation, with cell numbers adjusted according to the intended radiation dose: 250 and 500 cells for non‐irradiated controls; 750 or 1000 cells for 2 Gy; 1500 cells for 3–4 Gy; and 3000 cells for 5–6 Gy conditions. On the following day, cells were incubated with the AuNPs in complete DMEM for 4 h. Immediately before irradiation, the nanoparticle‐containing medium was removed, and 500 μL of fresh complete media was added to each well. Plates were then sealed with Mylar films and transported to the CNA for irradiation. The time from preparation to irradiation and return to the incubator is kept below 1 h. Irradiations were performed in a single fraction with doses ranging from 2 to 6 Gy. Only one of the two wells was irradiated, while the other served as a control, as significant radiation above background is not expected to reach it. This was previously verified experimentally by adding radiochromic films at this position for dose estimation measurements. Following exposure, the plates were returned to CABIMER. The Mylar film and medium were removed, and 2 mL of fresh complete medium was added to each well. Cultures were maintained for approximately 10 days to allow colony formation, with colonies reaching 50 cells. After 10 days of incubation, the samples were stained with crystal violet, and colony formation was quantified.

For quantification, the plates were scanned using an *Epson Perfection V850 Pro* scanner, and image analysis was performed with a custom macro developed in *ImageJ*. A minimum colony area threshold of 0.15 ${\mathrm{mm}}^{2}$ (corresponding to a radius of approximately 100 μm) was applied, as determined from microscopic observations. In addition, a circularity threshold of 0.3 was set, defined as $4\pi A/P^{2}$, according to the *Analyze Particles* function in *ImageJ*, where A represents the area of the object and P represents its perimeter, and the formula quantifies how close the shape is to a perfect circle.

### 2.9 Radiosensitization quantification

To quantify the effect of radiation, cell survival curves are generated using data from clonogenic assays. These curves describe the fraction of cells that retain the ability to proliferate and form colonies after irradiation, providing a quantitative measure of radiosensitivity. The Survival Fraction (SF) is computed as follows:

(1) $$\text{SF}=\frac{N_{I}}{N_{0}\times PE}\times 100,$$

where $N_{I}$ corresponds to the number of colonies after irradiation at a given dose, $N_{0}$ corresponds to the seeded cells and PE, the plating efficiency, calculated as the fraction of seeded cells that grow into colonies without any experimental intervention, and serves as a baseline to normalize survival after irradiation in the clonogenic assays.

(2) $$\text{PE}=\frac{N_{C}}{N_{0}}\times 100,$$

where $N_{C}$ corresponds to the number of colonies without irradiation.

The SF is a fundamental radiobiological parameter quantifying the proportion of cells retaining clonogenic potential after irradiation, serving as a critical endpoint to evaluate radiation efficacy and sensitizer performance. This is calculated for each condition (with and without AuNPs) and for each dose, and the resulting data subsequently fitted to the linear‐quadratic (LQ) model:

(3) $$\text{SF}=e^{-(\alpha D+\beta D^{2})},$$

where α and β are the model parameters. The parameter α is associated with irreparable complex damage and corresponds to the initial slope of the survival curve, while β represents damage from indirect effects and determines the curvature at higher doses (terminal slope). Note, that the overall cellular response depends on the radiation type as well as on the cell line. The LQ model interpretation relates higher α values to a stronger radiosensitization by increasing irreparable damage per unit dose.

The mean inactivation dose (MID) was introduced as a single parameter to characterize cell radiosensitivity, even in the case of curvilinear survival curves.^41^ This metric has been shown to provide more robust and consistent comparisons across repeated experiments and different cell lines. Although the MID does not capture the full complexity of survival‐curve shapes—reflecting instead the global cell response over the entire dose range—it is recommended by the ICRU^42^ for quantifying radiosensitivity and for comparing experiments involving radiosensitizing agents. The MID is defined as:

(4) $$\text{MID}=\int_{0}^{\infty }\text{SF}\left(D\right)dD,$$

where SF correspond to the survival curves defined in Equation (1). This approach represents the entire population, minimizes fluctuations in survival curves and takes into account the entire survival curve. The corresponding error is computed using the error propagation methodology taking into account the statistical errors of the model parameters. Then the Sensitizer Enhancement Ratio (SER) is computed as:

(5) $$\text{SER}=\frac{{\text{MID}}_{C}}{{\text{MID}}_{\text{NP}}},$$

where ${\text{MID}}_{C}$ corresponds to the MID of the survival curve without NP and ${\text{MID}}_{\text{NP}}$ to the survival curve with NPs. This observable describes the ratio of radiation doses required to achieve the same biological effect with and without NPs incorporating both physical dose enhancement and biological interactions. The associated error has been calculated using the error propagation method.

The amplification factor, AF, has also been computed to evaluate how the effect depends on the dose using the following equation:

(6) $$\text{AF}=\frac{{\text{SF}}_{\text{control}}-{\text{SF}}_{\text{NP}}}{{\text{SF}}_{\text{control}}}\times 100,$$

where SF stands for the survival fraction value defined in Equation (3).

### 2.10 Immunofluorescence and microscopy

Fluorescence microscopy was used to evaluate radiation‐induced DNA damage and repair through the counting of γH2AX foci (which identify double‐strand breaks, DSBs) and RPA foci to detect DNA end resection (the first step of the homologous recombination DSB repair pathway). This multiparametric approach allowed simultaneous visualization of DNA lesions and repair responses. For γH2AX and RPA foci visualization, HeLa cells were seeded on coverslips. At 1 h after irradiation (4 Gy), coverslips were washed once with PBS and then treated with pre‐extraction buffer (25 mM Tris–HCl, pH 7.5, 50 mM NaCl, 1 mM EDTA, 3 mM ${\mathrm{MgCl}}_{2}$, 300 mM sucrose, and 0.2% Triton X‐100) for 5 min on ice. Cells were fixed with 4% paraformaldehyde (w/v) in PBS for 20 min. Following two PBS washes, cells were blocked for 1 h with 5% FBS in PBS and co‐stained with the appropriate primary antibodies (for RPA: anti‐RPA32, Abcam (ab2175), 1:500 dilution; for γH2AX: anti‐γH2AX, Cell Signaling (2577L), 1:1000 dilution) in blocking solution overnight at 4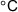 or for 2 h at room temperature. Cells were washed again with PBS and co‐immunostained with the corresponding secondary antibodies (for RPA: Alexa Fluor 594 goat anti‐mouse, Invitrogen (A11032), 1:1000 dilution; for γH2AX: Alexa Fluor 488 goat anti‐rabbit, Invitrogen (A11034), 1:1000 dilution) in blocking buffer. After washing with PBS and dehydration through 70% and 100% ethanol, coverslips were mounted onto glass slides using Vectashield mounting medium with DAPI (Vector Laboratories). RPA foci immunofluorescence was analyzed using a Thunder Imager microscope.

For quantification, ImageJ computer‐assisted scoring was used. Nuclei were selected by defining the ROI using DAPI staining. γH2AX intensity was measured, and foci were detected and quantified using a custom ImageJ macro (the Find Maxima tool). RPA‐positive cells (defined as cells containing 10 or more foci) were counted manually using ImageJ's cell‐counting tool.

## 3 STATISTICAL ANALYSIS

Data are presented as mean values ± standard error of the mean (SEM). Model fitting was evaluated using the R‐estimator, and the uncertainty associated with the SER parameter was determined by error propagation methods accounting for the full covariance matrix of the fit. Differences in the surviving fraction between groups treated with and without AuNPs were assessed using either a one‐way analysis of variance (ANOVA) or a Student's *t*‐test, implemented with Python statistical libraries or the *PRISM* software (GraphPad Software Inc.). A significance level of p<0.05 and *p* <0.01 was indicated for all statistical tests.

## 4 RESULTS

### 4.1 AuNP citotoxicity and internalization

**FIGURE 2:**
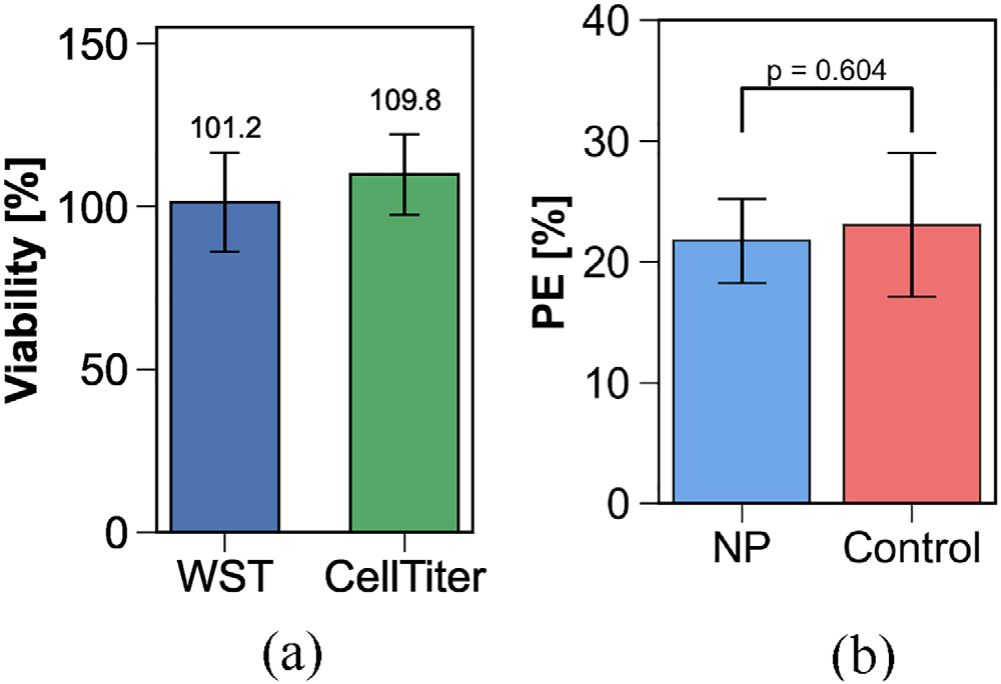
Assessment of cell viability. (a) Viability of HeLa cells measured using WST‐1 (colorimetric assay of mitochondrial activity) and CellTiter‐Glo^®^ (luminescence‐based ATP quantification) after 24 h incubation with 50 nm AuNPs (148 μg/mL). Results are expressed as percentage relative to untreated controls. Values represent the mean of multiple replicates (n=3). (b) Plating efficiency (PE) of HeLa cells incubated with AuNPs (148 μg/mL) for 4 h in the absence of irradiation. Colony formation was quantified 10 days post‐seeding and compared to untreated controls. Data represent the mean ± SD of multiple replicates (n≤3).

To optimize the experimental conditions, different AuNP dilutions in DMEM supplemented with FBS and various incubation times were tested. A 1:3 dilution, corresponding to 148 μg/mL incubated for 24 h, provided good stability, as the NPs remained well‐dispersed without precipitation and did not affect cell viability. Viability was assessed using two complementary assays: the CellTiter‐Glo^®^ Luminescent Cell Viability Assay, which measures ATP levels as an indicator of metabolic activity, and WST‐1, which evaluates mitochondrial function through the reduction of soluble tetrazolium salt. Both assays confirmed that AuNP exposure did not induce cytotoxic effects under the tested conditions (Figure 2a). To further evaluate the impact of AuNPs on cell proliferation, a clonogenic assay was performed under the same conditions used for irradiation experiments, but in the absence of radiation. Plating efficiency was calculated and compared to untreated controls. The results showed no significant difference between treated and untreated cells, indicating that nanoparticle exposure did not impair long‐term survival or proliferative capacity (Figure 2b).

**FIGURE 3:**
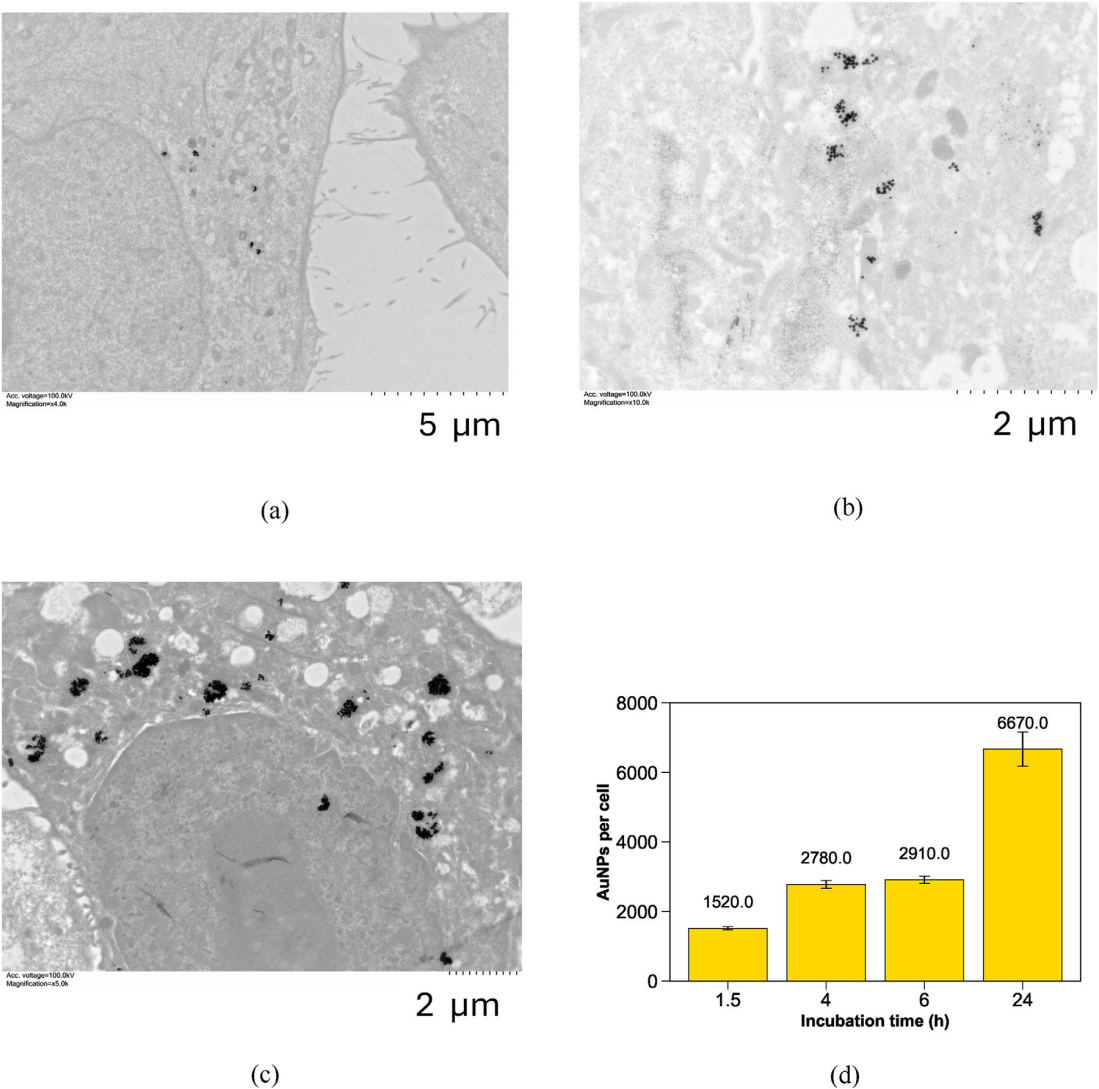
Quantification and localization of AuNP uptake in HeLa cells. (a, c) Representative images of internalization obtained with TEM showing AuNP internalization in HeLa cells after 4 h (a), 6 h (b), and 24 h (c) of incubation. (d) Quantification of AuNP uptake expressed as the number of nanoparticles per cell, measured by ICP‐MS at corresponding incubation times.

**FIGURE 4:**
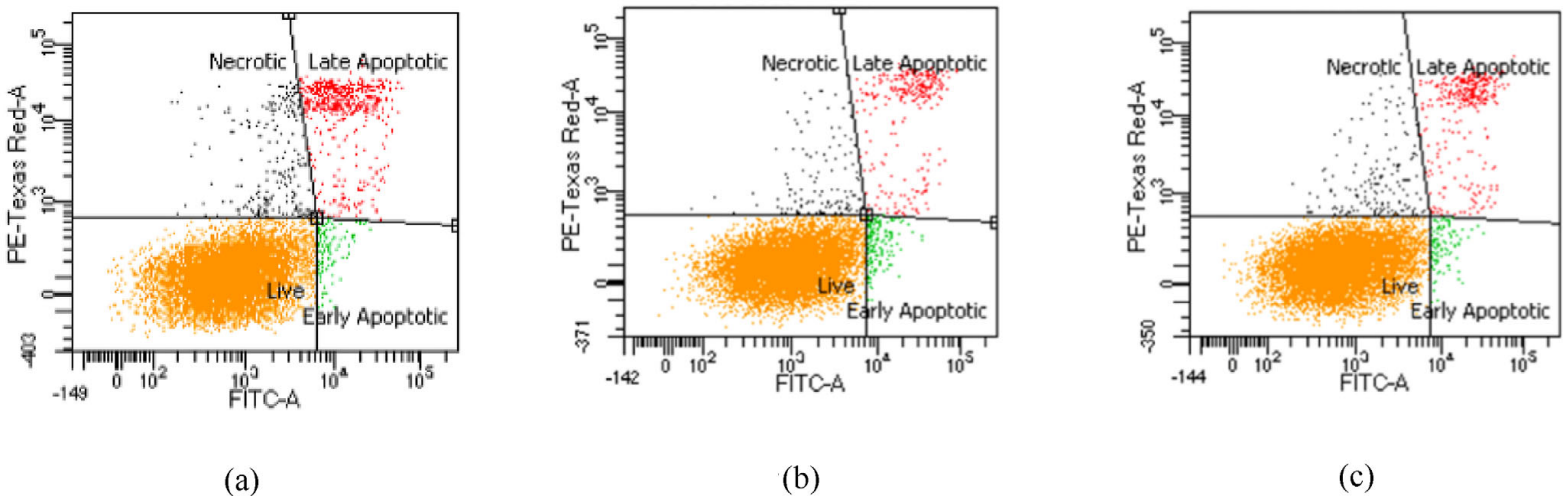
Assessment of cell viability and apoptosis following nanoparticle treatment. (a–c) Representative flow cytometry plots showing the distribution of viable (Annexin $V^{-}$/${\mathrm{PI}}^{-}$), early apoptotic (Annexin $V^{+}$/${\mathrm{PI}}^{-}$), late apoptotic/necrotic (Annexin $V^{+}$/${\mathrm{PI}}^{+}$), and necrotic (Annexin $V^{-}$/${\mathrm{PI}}^{+}$) cell populations after 0 h (a), 2 h (b), and 24 h (c) of incubation with 50 nm AuNPs.

To monitor nanoparticle uptake over time, the number of internalized AuNPs was quantified using ICP–MS (Figure 3d), and their intracellular localization was examined by TEM (Figure 3a–c). Quantitative analysis revealed a time‐dependent increase in nanoparticle internalization, with a marked accumulation between 6 and 24 h, reaching more than 6000 AuNPs per cell at 24 h (Figure 3d). This accumulation correlated with TEM observations, which showed a gradual enrichment of AuNPs within the cells. At 2 h, AuNPs were mainly observed in small, peripheral vesicular structures compatible with early endocytic compartments, often as individual particles. By 4 h, the AuNPs appeared in larger, more electron‐dense vesicles, which may indicate progression toward late endosomes or lysosomes. After 24 h of incubation, AuNPs were widely distributed throughout the cytoplasm, predominantly within vesicular compartments. Notably, while particles initially remained isolated, they gradually formed clusters, possibly reflecting their trafficking into more mature vesicles.

After 24 h of incubation, an increase in the number of intracellular vesicles was observed by TEM, which could indicate cellular stress. This feature has previously been associated with nanoparticle accumulation, although it does not necessarily imply cytotoxicity.^43^ To clarify whether this morphological change reflected cell damage, we performed a flow cytometry assay using Annexin V and propidium iodide. The results confirmed that apoptosis was not induced under these conditions, supporting the notion that AuNP exposure did not compromise cell viability (Figure 4a–c and data in Table 1).

**TABLE 1:** Apoptosis analysis by flow cytometry using the FITC–Annexin V/PI staining kit; results represent the average of three wells per condition.

Time	Live	Early apoptosis	Late apoptosis	Necrosis
0	90.8 ± 2.5	1.6 ± 0.1	4.4 ± 0.1	3.2 ± 2.4
2h	89.5 ± 6.5	1.0 ± 0.3	6.3 ± 5.0	2.2 ± 1.7
4h	88.2 ± 1.2	1.7 ± 1.1	7.5 ± 2.0	2.8 ± 1.0
24h	93.2 ± 1.8	2.6 ± 0.3	3.6 ± 2.0	0.5 ± 0.1

Based on these observations, and considering practical aspects of the experimental setup, a 4‐h incubation with NPs was selected as the optimal condition. This duration ensured sufficient NP uptake without affecting cell viability and was therefore used in subsequent irradiation experiments.

**FIGURE 5:**
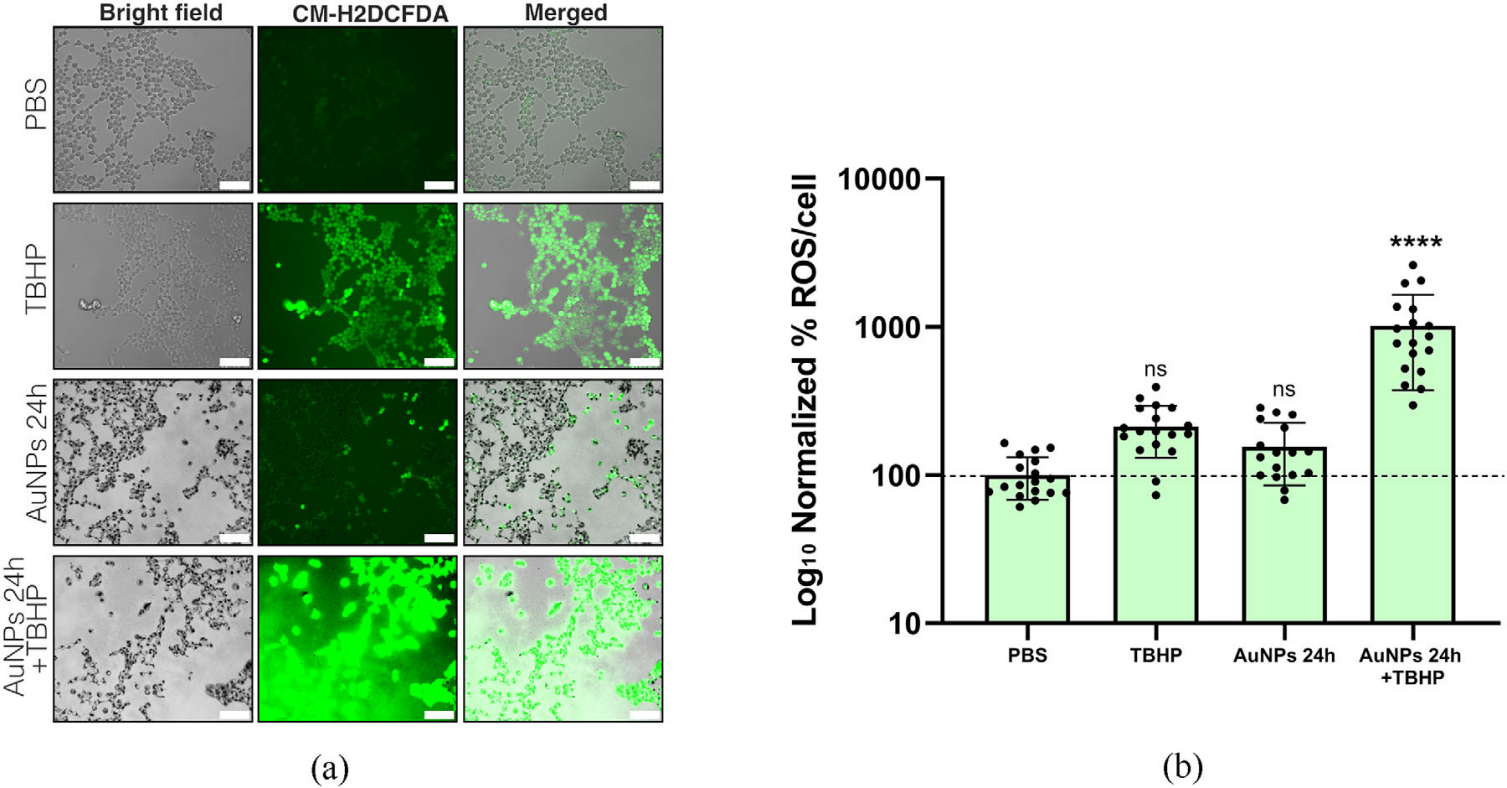
Analysis of intracellular oxidative stress properties of AuNPs by $\text{CM}-H_{2}\text{DCFDA}$ fluorescence. (a) Representative bright‐field and fluorescence images after both untreated (PBS) HeLa or treated with TBHP for 1h, AuNPs for 24h, or AuNPs for 24h + TBHP for 1h. Images were acquired after incubation with $\text{CM}-H_{2}\text{DCFDA}$. Scale bar=100μm. (b) Relative fluorescence units for the intracellular $\text{CM}-H_{2}\text{DCFDA}$ normalized to the number of viable cells. The mean and error bars are shown. Solid dots represent the results of individual measurements (technical replicates). Untreated HeLa cells (PBS) incubated with $\text{CM}-H_{2}\text{DCFDA}$ were used as a 100% reference for normalization across three biologically independent experiments (dashed black line). Statistical analysis was performed using one‐way ANOVA comparing each condition to this control. ns: not significant; **** *p* <00001.

To investigate if AuNPs themselves contribute to oxidative stress, intracellular ROS levels were measured by $\text{CM}-H_{2}\text{DCFDA}$ fluorimetry and visualised by fluorescence microscopy in cells incubated with AuNPs (Figure 5). AuNP‐treated cells showed no significant increase in ROS compared to untreated controls, indicating that AuNPs alone do not induce oxidative stress under basal conditions. Additionally, when cells were exposed to TBHP after incubation with AuNPs, ROS levels were significantly higher than in cells treated with TBHP alone (Figure 5), suggesting that AuNPs can amplify ROS production when an oxidative stimulus is present.

### 4.2 Proton beam homogeneity and dose verification

**FIGURE 6:**
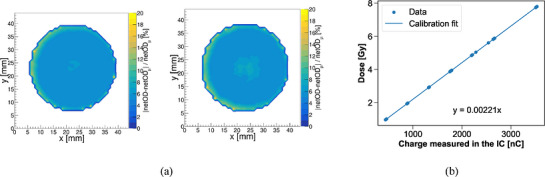
Beam homogeneity characterization and dose calibration. (a) Two‐dimensional maps of dose homogeneity for the proton beam, measured with radiochromic films prior to irradiation. The color scale represents the relative deviation of the local net optical density from the mean value. (b) Calibration curve correlating the charge measured in the ionization chamber with the dose delivered to the cells, obtained from Monte Carlo simulations for the final experimental configuration

As introduced in the first section, the beam homogeneity is measured every day before starting the biological samples irradiations. Figure 6a shows two examples of two‐dimensional maps of dose homogeneity obtained for the 2022 experimental campaign. The colour scale represents the relative deviation of the local net optical density from the mean value, expressed as a percentage. Both irradiations display a highly uniform central region, with deviations below 5% across most of the field, while slightly larger variations are observed at the beam edges, reaching values of 15%–20%. In all irradiations, these measurements were systematically performed at the beginning and the beam adjusted to provide homogeneity levels around 5%. This highlights the reproducibility of the beam delivery and confirms the suitability of the experimental setup to provide a homogeneous proton irradiation field within the region of interest.

As described in the previous section, a new calibration for dose measurements was performed to account for the effect of the Mylar film added to the experimental setup in comparison to previous tests. The presence of the Mylar film was estimated to reduce the kinetic energy of the incident protons by 3.6% compared to the previous calibration. The resulting calibration curve is shown in Figure 6b. The influence of cell layer thickness was evaluated by performing simulations for 20 and 40 μm, revealing a variation of only 0.1%, which was considered negligible in terms of the average LET. Under these conditions, the mean kinetic energy of the protons reaching the cell culture is 12.6 MeV. Irradiations were performed at a dose rate of 4 Gy/min.

### 4.3 Radiosensitization effect measurements

**FIGURE 7:**
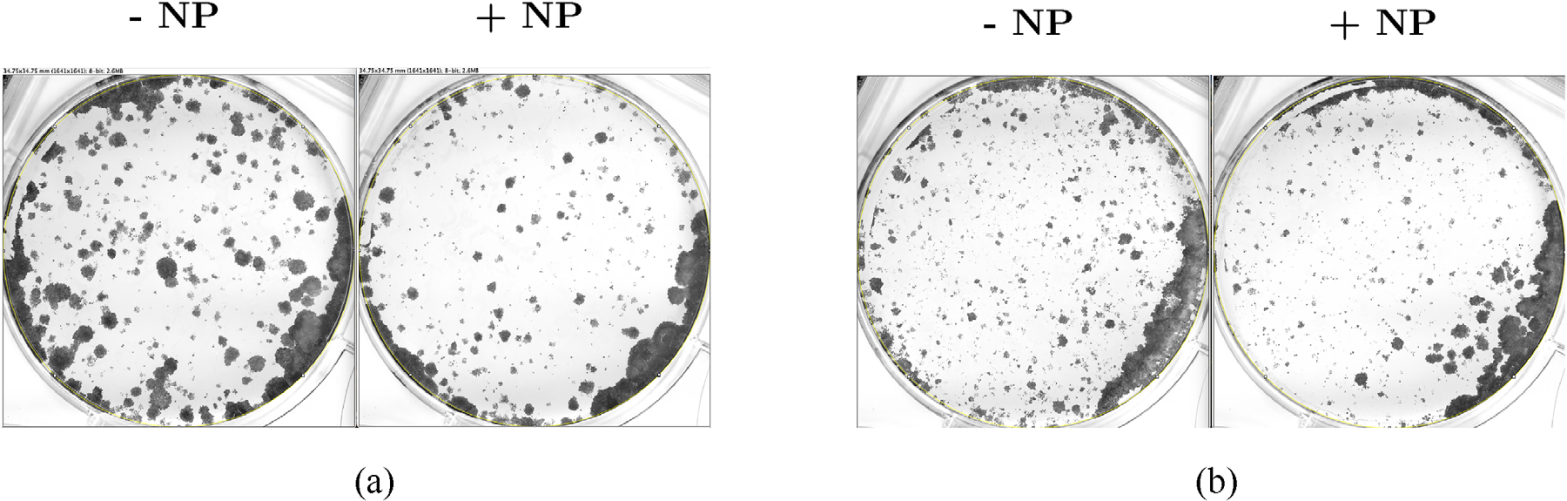
Representative clonogenic survival plates with and without AuNPs. Representative images of the clonogenic assays after irradiation either mock treated (‐) or treated with AuNPs (+). After 4 Gy (a) and 5 Gy (b) of irradiation.

The first approach used to quantify the radiation effect with and without AuNPs is based on the analysis of clonogenic assays. Figure 7 shows representative colony plates for 4 Gy (A) and 5 Gy (B) without AuNPs (‐NP) and with AuNPs (+NP). The region of overgrowth corresponds to the area of the well where the medium accumulates when the plate is positioned vertically. In this section, protons are stopped by the excess medium, preventing irradiation of the underlying cells. This feature serves as a clear indicator that the irradiation was correctly performed. We can also observe that, in the control condition (left), colonies are more numerous and generally larger than in the AuNP‐treated sample (right).

**FIGURE 8:**
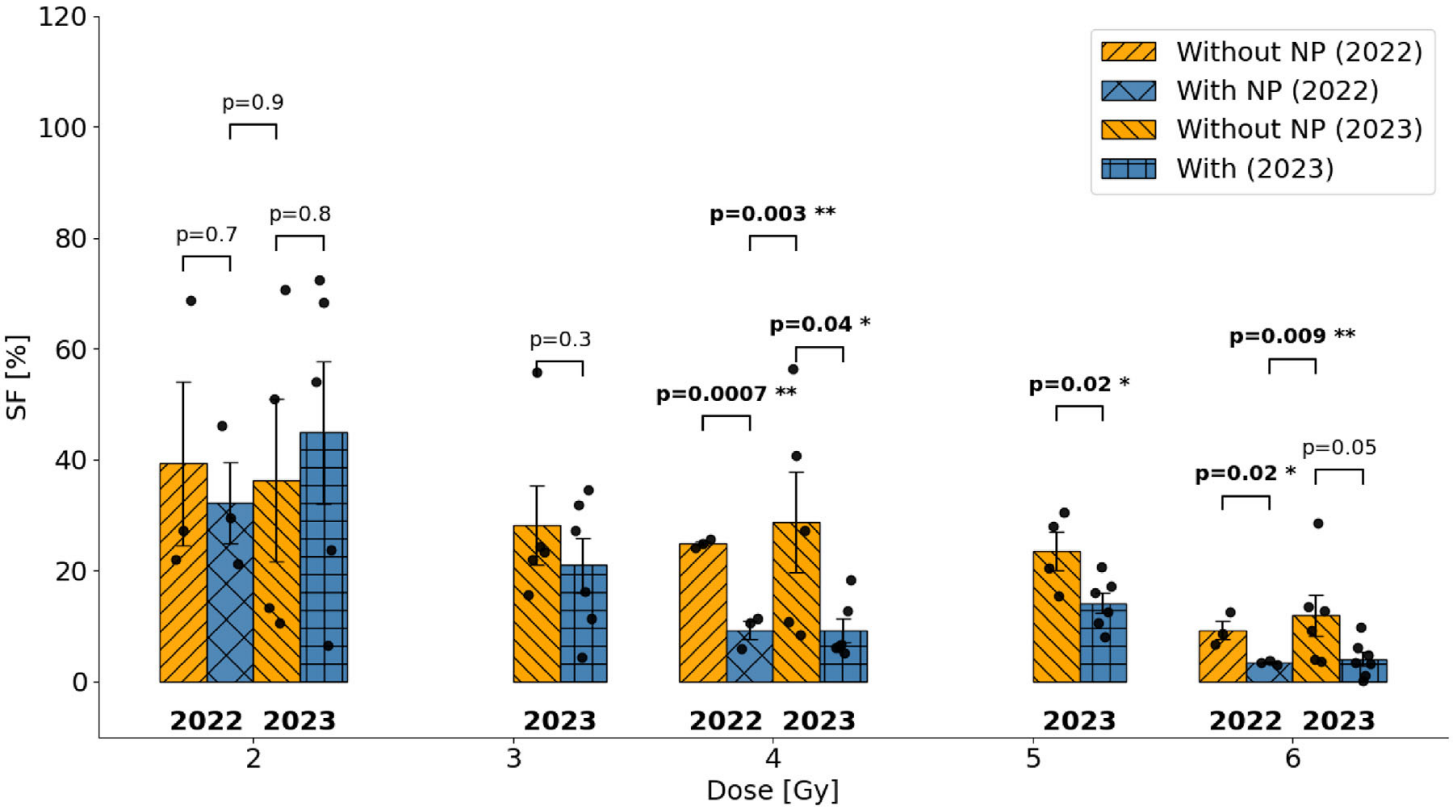
Survival Fraction of HeLa cells irradiated with protons in the presence and absence of AuNPs at different doses for the 2022 and 2023 experimental campaigns. Bars represent the mean ± standard deviation from independent replicates, while individual data points correspond to measured values for each condition. Note that the dose displacements has been performed artificially for visualization porpoise. Orange bars indicate samples without AuNPs and blue bars those incubated with AuNPs. Statistical significance between paired conditions is shown, with p<0.05 and p<0.01 indicated by one and two asterisks, respectively.

The colonies of all samples were counted, and the plating efficiency (PE) and survival fraction (SF) were calculated as described in Equations. (1) and (2). Figure 8 shows the SF of HeLa cells as a function of proton dose for two independent experimental campaigns conducted in 2022 and 2023. In both campaigns, the mean cell survival generally decreases with increasing dose, as expected. The presence of AuNPs consistently reduces the SF across most doses, indicating a radiosensitizing effect. This effect is statistically significant at 4, 5, and 6 Gy (p<0.05, p<0.01), while at 3 Gy a similar trend is observed but does not reach statistical significance. No radiosensitizing effect is detected at 2 Gy.

Comparison between campaigns demonstrates good reproducibility, with consistent trends across years, although the 2023 data show slightly larger fluctuations. In 2022, only three replicates per dose and condition were irradiated on the same day. In contrast, the 2023 campaign included five replicates for the control group (without AuNPs) and five to six replicates for AuNP‐treated samples, depending on the dose, distributed over three different days. Due to these logistical constraints, data from different days had to be combined, which could increase the variability in the 2023 dataset. Nevertheless, despite the larger associated uncertainties, the results remain consistent with those from 2022 and confirm the radiosensitizing effect of AuNPs.

**FIGURE 9:**
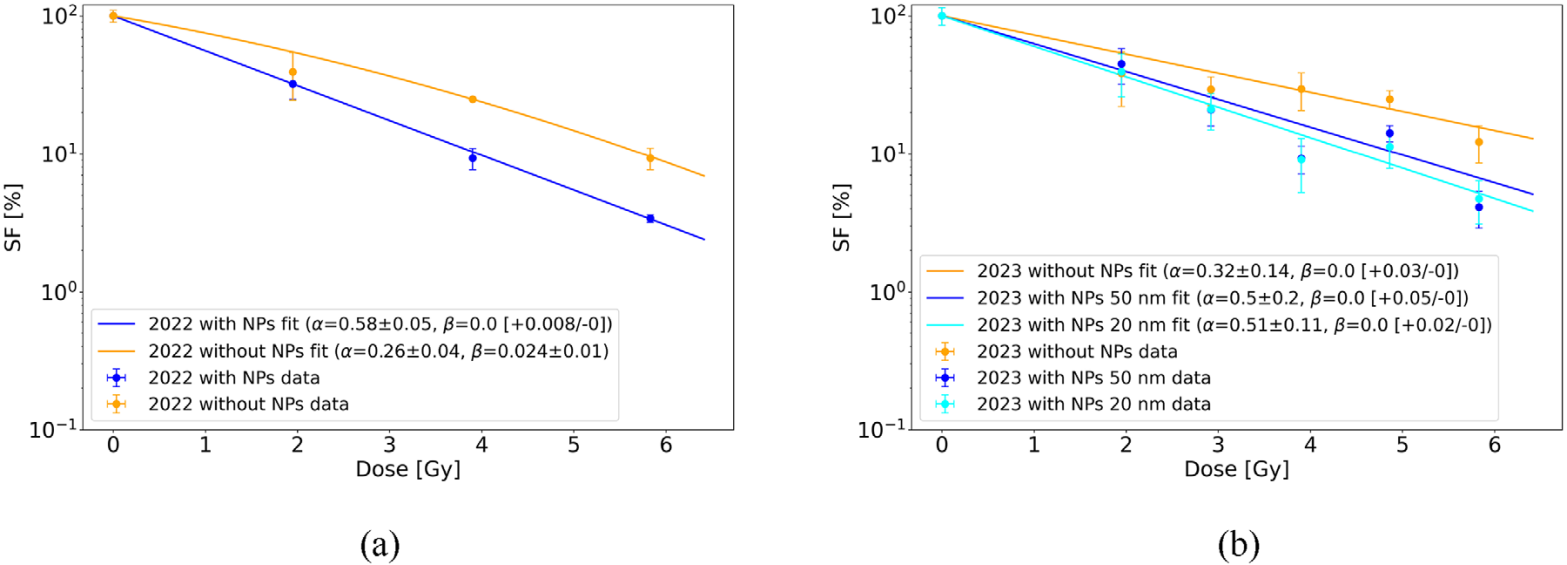
Survival Fractions (SF) of HeLa cells irradiated with protons in the absence and presence of AuNPs, fitted using the LQ model. (a) 2022 experimental campaign with 50 nm AuNPs. (b) 2023 experimental campaign including results for both 20 and 50 nm AuNPs. Each data point represents the mean SF ± standard error. The fitted parameters (α and β) are indicated in the legend.

Figure 9 shows the survival curves obtained with the 2022 (Figure 9a) and 2023 (Figure 9b) data fitted using the LQ model. In both campaigns, cells incubated with AuNPs exhibited a steeper decline in SF compared to control cells without nanoparticles as reflected by the higher α parameter and reduced survival at all doses. In 2023, additional experiments compared 20 and 50 nm AuNPs effect, both showing similar radiosensitization trends, suggesting that nanoparticle size within this range has a limited influence on the overall effect. In all cases a near‐zero value for β was obtained.

Table 2 summarizes the LQ fitting parameters. For the 2022 dataset, the control condition (without AuNPs) exhibits an α value of 0.26 and a non‐zero β component. In contrast, the sample containing 50 nm AuNPs shows a markedly higher α value (0.58) and a negligible β, suggesting that the presence of nanoparticles enhances the probability of irreparable DNA damage. The corresponding SER of 1.53 confirms a substantial radiosensitizing effect of the 50 nm AuNPs under these conditions.

**TABLE 2:** LQ fitting summary includes the fitted α and β parameters, their corresponding statistical errors, the goodness of the fit R‐value, and the SER value calculated relative to the control (without AuNPs).

AuNPs	α	β	*R*‐value	SER
Control 2022	0.264 [‐0.039,0.039]	0.024 [‐0.01,0.01]	0.99	1
AuNP 2022 50 nm	0.581 [‐0.048,0.048]	0.00 [0,0.008]	0.95	1.53 [‐0.07,0.07]
Control 2023	0.322 [‐0.135,0.135]	0.00 [0,0.028]	0.97	1
AuNP 2023 50 nm	0.454 [‐0.213,0.213]	0.00 [0,0.044]	0.86	1.3 [‐0.2,0.2]
AuNP 2023 20 nm	0.51 [‐0.11,0.11]	0.00 [0,0.02]	0.88	1.39 [‐0.18,0.18]

In 2023, similar trends were observed despite a slightly larger data variability. The control condition yielded an α of 0.32 and β of zero value, while both AuNP‐treated cases (20 and 50 nm) showed increased α values. The β term remained negligible across all irradiations. The SER values (1.30 for 50 nm AuNPs and 1.39 for 20 nm AuNPs) confirm the persistence of the radiosensitizing effect and suggest no significant size dependence within the tested range being both cases compatible with the 2022 data.

**FIGURE 10:**
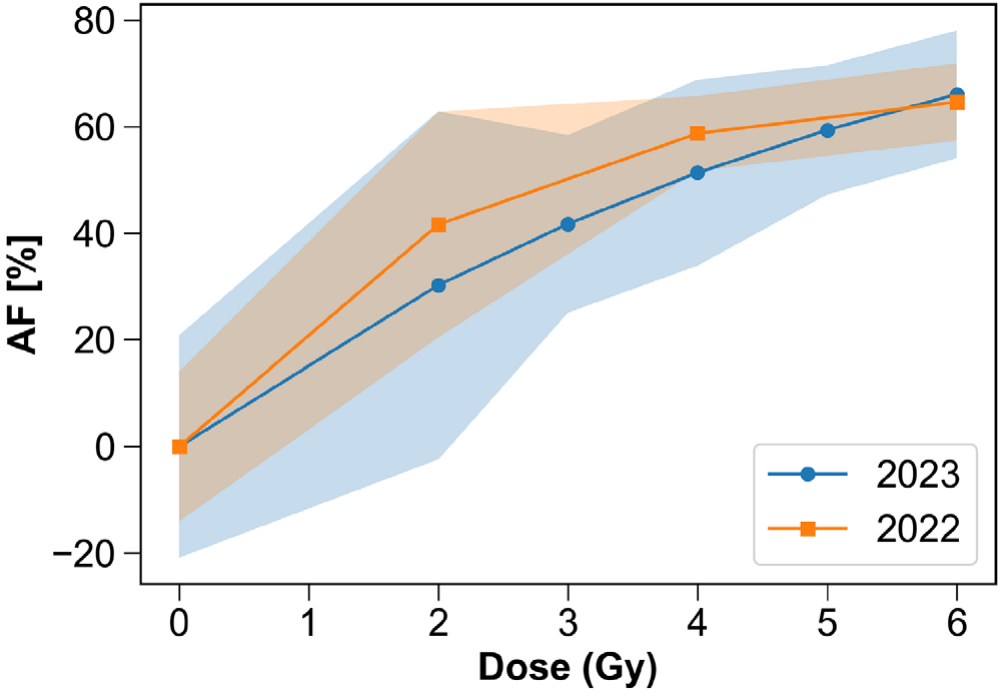
Amplification factor computed for the data of the two experimental campaigns as a function of the proton dose. The amplification factor (AF) quantifies the relative decrease in cell survival cause by the presence of AuNPs and is obtained by comparing, at each dose, the difference between the survival fraction of control cells and AuNP treated cells, normalized to the control value. Shaded bands represent the associated uncertainties obtained through error propagation.

Finally, the amplification factor, which quantifies the relative reduction in cell survival due to the presence of AuNPs compared with the control cells, was also calculated for each dose (Figure 10). In both experimental campaigns, this factor increases with dose, reaching a maximum value of approximately 60% at 6 Gy. The associated uncertainties, computed through error propagation, are represented by a shaded band. Within their respective uncertainties, the results from the two campaigns are consistent.

### 4.4 Double strand break assays

**FIGURE 11:**
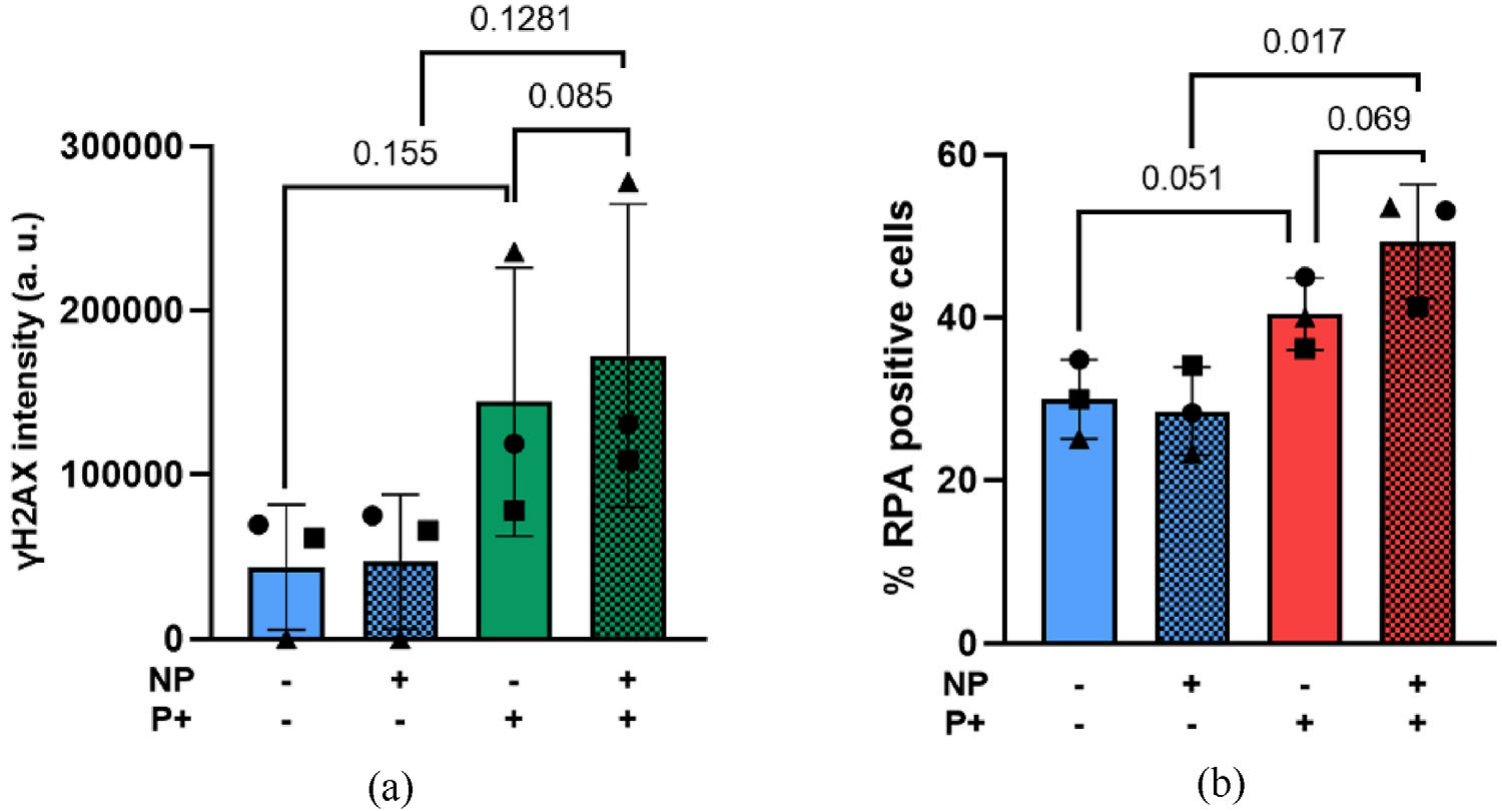
Radiation‐induced DNA damage signalling and repair in the presence or absence of gold nanoparticles. HeLa cells were incubated for 4 h with AuNPs or mock‐treated, irradiated with 4 Gy protons, and fixed 1 h later for immunofluorescence detection of DNA damage markers: DSBs (γH2AX) and early homologous recombination repair (RPA foci). Mock‐treated, irradiated and non‐irradiated cells were included as controls. The experiment was independently repeated three times. (a) γH2AX quantification (γH2AX fluorescence intensity per cell). Foci quantification was performed using automated computer scoring (see the methods section). (b) RPA‐positive cell quantification, that is percentage of RPA‐positive cells (defined as cells containing more than 10 RPA foci). Foci quantification was performed using manual scoring of the immunofluorescence images. In all cases, data represent the mean ± SD from three independent experiments and statistical significance was determined with a Student's t‐test.

**FIGURE 12:**
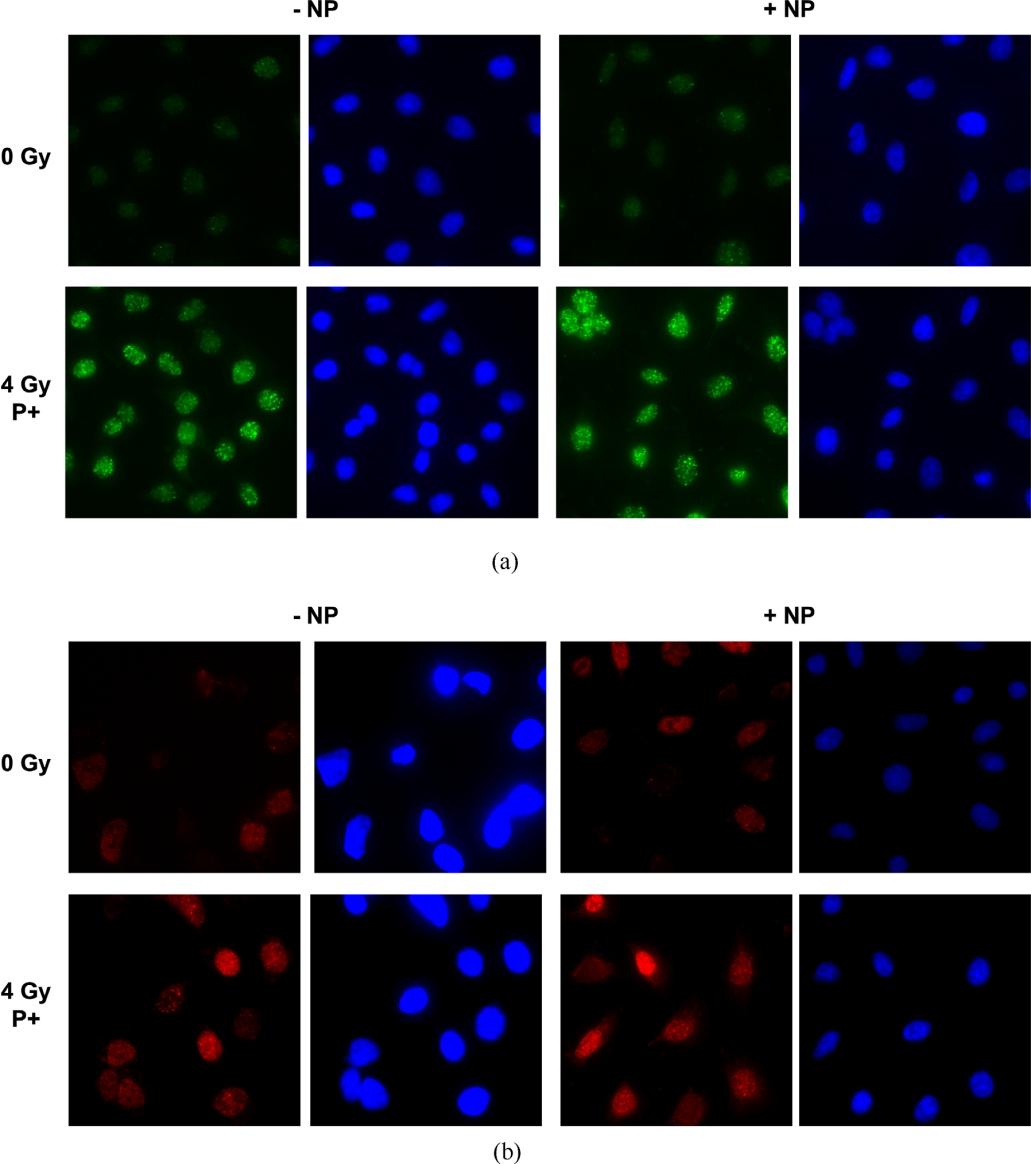
Representative fluorescence microscopy images of DNA damage and repair markers in HeLa cells. Images show γH2AX foci (green) (a) and RPA‐positive cells (red) (b), together with DAPI‐labelled nuclei. Both AuNP‐treated (+) and mock‐treated (−) cells are shown, including non‐irradiated controls and cells irradiated with 4 Gy protons.

To further investigate the biological contribution of AuNPs to radiosensitization, we assessed their impact on DNA double‐strand break (DSB) signaling and early repair processing. γH2AX and RPA foci accumulation were selected as complementary markers: γH2AX reflects the presence of DSBs, whereas RPA foci indicate DNA end resection, the first step of the homologous recombination repair pathway. After 4 h of treatment with AuNPs, cells were exposed to 4 Gy proton irradiation and then incubated for an additional hour to allow DNA damage sensing and the initiation of DNA resection. Immunofluorescence experiments were performed to quantify the accumulation of γH2AX and RPA at DSB sites. As can be seen in Figure 11a, treatment with AuNPs increased DNA damage accumulation (measured as the overall fluorescence intensity), only following proton irradiation. In addition, Figure Figure 11b shows DNA resection efficiency (measured as the percentage of RPA foci‐positive cells) for the same experimental conditions. Here, an increase in DNA end resection was observed after 4 Gy proton irradiation, with a stronger effect in cells treated with gold nanoparticles. Representative fluorescence microscopy images corresponding to these analyses are shown in Figure 12a (for γH2AX) and Figure 12b (for RPA).

### 4.5 Robotic system for improved experimental procedure

**FIGURE 13:**
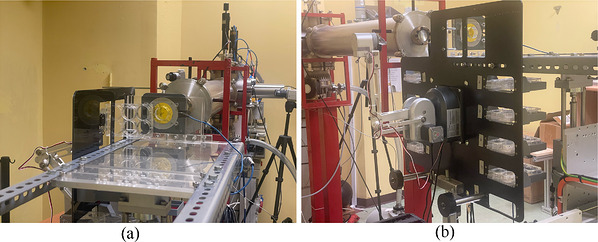
Robotic Platform for Automated Transfer of Cell‐Culture Plates in Radiobiology Workflows. (a) Robot system tested at CNA external cyclotron beam line for radiobiology experiments. (b) A 6‐well plate being placed in the beam path for irradiation with the robotic system.

The increased variability observed in the 2023 experiments highlighted the need to minimize the time that cell‐culture plates remain vertical without medium and outside the incubator. To this end, a robotic arm equipped with a custom 6‐well‐plate gripper and a dedicated support frame with staging shelves was developed in collaboration with Vicent Girbés from the *Escuela Técnica Superior de Ingeniería, Universitat de València* and the company *Mecanismos Técnicos y de Laboratorio S.L*. . The system moves cell‐culture plates sequentially between resting and irradiation positions (Figure 13), enabling automated irradiation workflows for radiobiology experiments and improving both efficiency and experimental conditions.

Although this system was not used for the data presented here, it was developed in the context of these experiments and commissioned in 2025 (Supplementary Material: Robotic arm video). Initial tests demonstrated that the system allows for the irradiation of six samples in approximately 20 min, compared with 1.5 h required when manual handling is performed, which involves repeated individual transfers and waiting periods for radiation decay (8 min) before accessing the irradiation bunker. This improvement allows a higher number of replicates to be processed per day under more controlled conditions, enhancing reproducibility and experimental throughput.

## 5 DISCUSSION

The combination of proton therapy, which inherently spares surrounding healthy tissue, with the radiosensitizing potential of AuNPs offers a promising strategy to further enhance the clinical efficacy of proton therapy. Although many mechanistic aspects remain to be elucidated, the initial biological findings are encouraging and strongly motivate further investigation into the synergistic interactions between proton irradiation and AuNPs. In this study, cells were incubated for 4 h with spherical, unfunctionalized, 50 and 20 nm AuNPs prior to irradiation. Under the tested conditions, the AuNPs did not induce detectable cytotoxicity or an increase of ROS basal levels for incubation times up to 24 h, confirming that the nanoparticles themselves do not act as primary toxic agents in the absence of irradiation. Additionally, we characterized the cellular uptake of the nanoparticles. ICP‐MS quantification revealed a time‐dependent internalization, with a few thousand AuNPs per cell after 4 h, while TEM imaging showed that the AuNPs were efficiently internalized and predominantly localized in cytoplasmic clusters rather than in the nucleus.

From the radiobiology experiments presented in this article a SER ranging between 1.3 and 1.5 is observed, consistent with values reported in the literature for similar AuNP‐based proton irradiation experiments included in Table 3. The radiosensitizing effect becomes more evident and statistically significant at doses above 4 Gy, while it is less pronounced at 2 and 3 Gy. This reduced effect at lower doses may be partly attributed to the low plating efficiency of the cell line used, measured around 10–20%, which results in a smaller effective number of seeded cells than expected. Combined with the lower probability of interactions between protons or secondary particles and the AuNPs at low doses, this may lead to increased variability in the clonogenic survival data at these dose levels. As the dose increases, the number of seeded cells used also increases (e.g., approximately 1000 cells at 2 Gy, 1500 at 3–4 Gy, and 3000 at 5–6 Gy), thereby enhancing the statistical robustness of the measurements. Moreover, when analysing the AF as a function of dose, a clear upward trend is observed, in agreement with previous reports. This trend suggests that the radiosensitizing effect becomes more pronounced at higher doses.

**TABLE 3:** Summary of AuNP radiosensitization studies with protons, including NP size, concentration/uptake, incubation time, and observed effect.

Year	Cell	Energy [MeV/n]	NP	Size [nm]	Conc./Uptake	Time [h]	Effect
2010^22^	EMT‐6	3	AuNP‐PEG	6.1 ± 1.9	0.1 mL of 500 μM Au	48	↓ in SF 2–11.9%
	CT26				—		
2011^14^	DU145	160 (SOBP)	Citrate‐AuNPs	44 ± 8	—	—	↑ ∼ 15–20% RBE
					1 ng/cell		
2012^15^	Mouse	40	Au and Fe NPs	14 ± 1.2	—	—	↑ tumor control
	CT26			10.6 ± 0.8	41 μg Au/g		
					59 μg Fe/g		
2014^17^	RT112	3	AuNPs	50	—	4	No significant effect
					5000 NPs/cell		
2015^44^, *	Hela	165	Citrate‐AuNPs	15	1.0–15 μg/ml	24	SER = 1.27–1.44
					—		(depending on conc.)
2016^18^	A431	1.3 and 4	AuNPs	5, 10	0.05–0.10 mg/ml	24	
					0.30–0.78 pg Au/cell		
2018^23^	ALTS1C1	200 (SOBP)	AuNPs–PEG	1.8–2.6	8.75–140 μg/ml	24	SER = 1.32–1.44
			–cRGDs		—		
2019^20^	HCT116	150	AuNPs, SPIONs,	1.9, 15	—	—	ROS enhancement
			PtNDs, BiNRs	42, 70	—		
2021^25^	CHO‐K1	∼200 (SOBP)	AuNPs	50	10 μg/mL	4	AF(2 Gy) = 27.1%
					0.89 ± 0.044 pg/cell		AF(6 Gy) = 43.8%
							No LET effect
2023^35^	A431	230 (SOBP)	AuNPs	55	—	24	AF(2 Gy) = 17.6%
							AF(4 Gy) = 23.49%
					134 pg/cell		AF(6 Gy) = 33.99%
							ROS enhancement
2025^45^	HCT 116	150	AuNPs	1.9	3 mM/L	24	SER(50%) = 2.18
							SER(70%) = 2.52
					—		SER(90%) = 3.78
							ROS enhancement

* Carbon beam; arrows indicate increase (↑) or decrease (↓) in survival fraction (SF).

Experiments with 20 nm AuNPs conducted in the 2023 campaign produced a SER of 1.38 ± 0.18, showing no significant difference compared to 50 nm particles. This indicates that both sizes elicit a similar radiosensitizing effect.

### 5.1 Comparison with previous studies

Several experimental studies have demonstrated the radiosensitizing potential of different types of AuNPs under a variety of proton beam conditions.^14^, ^15^, ^16^, ^17^, ^18^, ^19^, ^20^, ^21^, ^22^, ^23^, ^24^, ^25^ However, the variability in experimental setups makes it challenging to directly compare or integrate results and draw firm conclusions regarding the influence of nanoparticle properties, including the impact of linear energy transfer (LET). Although the present study was conducted at a proton energy different from that used in clinical beams, it remains relevant. This is partly because the selected energy lies within the Bragg peak region and partly because there is still no clear consensus on how LET affects the radiosensitizing effect of nanoparticles. In this work, we have expanded the table previously presented by Cunningham et al.^25^ (Table 3) to include a more comprehensive list of experimental parameters, providing a broader context for interpreting and comparing results.

The studies summarized in Table 3 illustrate the current landscape of proton‐based AuNP radiosensitization experiments. A wide range of particle sizes has been investigated, from 1.8 to 70 nm, with different surface modifications and compositions, including PEGylated AuNPs, citrate‐capped particles, and no coated AuNPs. An important aspect of the present work is the direct comparison between two nanoparticle sizes, 20 and 50 nm diameter. While a large body of literature has focused on a limited range of nanoparticle sizes, systematic comparisons under identical irradiation and biological conditions with protons remain relatively scarce. This comparison therefore provides additional insight into the role of nanoparticle size within relevant sizes for its future applicability. This is motivated not only by physical considerations related to interaction probability and dose enhancement, but also by biological and translational aspects such as tissue penetration, cellular uptake, and selective targeting strategies, which often require complex and costly peptide functionalization. While 50 nm AuNPs have been reported to achieve efficient uptake in cell monolayers, smaller nanoparticles (20 nm) exhibit superior uptake and deeper penetration in more complex three‐dimensional structures tumour models.^46^

Irradiation energies span from a few MeV up to clinical SOBP ranges ( 200 MeV), with both low‐LET and higher‐LET conditions explored. First, it is worth noting that at low proton energies (1.3 and 4 MeV), corresponding to very high LET conditions, the reported radiosensitization effect in the literature is generally weak, absent, or not quantified with SER values, regardless of cell line, nanoparticle size, or concentration. In contrast, the present results obtained at 12.6 MeV exhibit SER and AF values comparable to those reported in higher‐energy and spread‐out Bragg peak proton studies (160–230 MeV). This observation suggests that the intermediate energy regime explored in this work may be sufficient to effectively probe nanoparticle‐induced radiosensitization in in vitro studies, while offering a more accessible and controllable experimental configuration that does not require clinical proton beam facilities. Assuming a limited dependence of the radiosensitization effect on beam energy within the Bragg peak region, the most directly comparable study is,^25^ which employs similar nanoparticle size, incubation time, and intracellular gold concentration. Within the associated experimental uncertainties, the reported AF are similar between both studies, although differences in cell lines should be considered when interpreting the comparison.

Another important observation is the increase of the amplification factor with delivered dose, which supports the relevance of physical interaction mechanisms in nanoparticle‐enhanced proton irradiation. At higher doses, the probability of interactions between incident protons, as well as secondary particles, and the nanoparticles is expected to increase, leading to a greater likelihood of localized energy deposition events. This behaviour is consistently observed in studies by Cunningham et al. and Lo et al.^25^, ^35^ and in the present work. Quantitatively, for an increase in dose of approximately a factor of three from 2 to 6 Gys, the corresponding gain in amplification is within the range of 1.6–2.2 in this study, 2.1 in,^25^ and 1.9 in.^35^ Notably, these studies involve different cell lines, which may contribute to variability in the biological response. In addition, while our work and^25^ report similar nanoparticle concentrations per cell,^35^ employed concentrations up to two orders of magnitude higher, yet without a proportionally larger amplification effect. This suggests that the relationship between dose, nanoparticle concentration, and radiosensitization is not simply linear. One possible explanation is the occurrence of nanoparticle clustering, as observed in TEM images, which has been shown in Monte Carlo simulations^27^ to reduce overall effectiveness due to the reabsorption of secondary electrons within nanoparticle aggregates.

Overall, these results demonstrate that AuNPs can enhance proton‐induced cytotoxicity in vitro, but the effect strongly depends on experimental parameters, emphasizing the importance of standardized protocols for future research.^42^ A further strength of this work lies in the comprehensive and quantitative characterization of the radiosensitization effect. In addition to survival curves, the study reports systematically derived metrics such as the SER, MID and AF, accompanied by a full uncertainty analysis. This level of quantitative description is still relatively limited in the literature, where many studies focus on qualitative trends or single‐dose comparisons. A more rigorous and systematic characterization is essential for enabling meaningful inter‐study comparisons and for supporting the development of predictive models of nanoparticle‐mediated radiosensitization.

### 5.2 Mechanistic insights into AuNP radiosensitization

Regarding DNA damage accumulation, and in agreement with the viability results after irradiation, an increase in DNA damage at 4 Gy is observed in the presence of AuNPs, in terms of γH2AX or RPA foci accumulation. DNA damage induced in the presence of nanoparticles can arise from multiple, potentially concurrent mechanisms spanning the physical, chemical, and biological stages of radiation action. These processes ultimately lead to a spectrum of DNA lesions, including single‐strand breaks (SSBs), double‐strand breaks (DSBs), base damage, mutations, and chromosomal aberrations, which may culminate in cell death if not properly repaired.^47^

At the physical level, interactions of primary and secondary particles, produced by the radiation impacting the AuNPs, may contribute to direct and indirect DNA damage. In order to quantify the physical contribution to radiosensitization effect of AuNPs Monte Carlo simulations are used. The dose enhancement factor (DEF), defined as the ratio of the deposited dose in the presence and absence of NPs under identical irradiation conditions, is calculated as a measure of the purely physical contribution to radiosensitization. Its magnitude is strongly dependent on the scoring volume and geometrical configuration. DEF values ranging from 1.7 to more than one order of magnitude in the immediate vicinity of the AuNP surface have been reported for proton beams interacting with 50 nm AuNPs.^27^, ^28^, ^29^ However, when more realistic irradiation conditions are considered including: increased distances between the proton tracks and the NP surface, AuNP aggregation, the spatial distribution of AuNPs within the medium, and the probabilistic nature of particle interactions within a full beam, these local enhancements are significantly reduced and may become negligible at biologically relevant scales. In contrast, the experimentally observed radiosensitization effects remain significant under such conditions. This discrepancy suggests that physical dose enhancement mechanisms are not sufficient to fully explain the observed biological response and supports the need to consider additional chemical and biological contributions.

At the chemical level, it is known that low‐LET beams cause indirect DNA damage through ROS production by water radiolysis.^48^ In this work, we demonstrate that AuNPs significantly enhance ROS production under oxidative conditions. Therefore, during proton irradiation, where an oxidative environment is generated through the interaction of protons with water molecules, AuNPs may contribute to amplifying the ROS induced and damage the DNA and affect the cell viability. In addition, at the biological level, secondary amplification mechanisms cannot be excluded, including mitochondrial dysfunction and redox imbalance induced by AuNP uptake which may further enhance ROS‐mediated damage and compromise DNA repair efficiency. Together, all these effects may contribute to the increased levels of DNA double‐strand breaks and surviving fraction curves observed under irradiation in the presence of nanoparticles.

From a mechanistic perspective, an important next step will be to further clarify and quantify the underlying processes responsible for the observed reduced viability and increased DNA damage. In particular, it would be relevant to confirm whether ROS production is a dominant contributor to the measured effects and to further investigate the downstream pathways leading to DNA damage. In this context, complementary approaches such as proteomic analyses could provide valuable insight into the cellular response, including stress‐related pathways, DNA repair modulation, and potential mitochondrial involvement, helping to better resolve the biological origin of the observed radiosensitization. At a longer‐term level, one limitation of the present study is the use of a single cell line and a simplified 2D monolayer culture model, which does not fully capture the biological complexity of tumors. Future studies should therefore extend these investigations to additional cell lines with different radiosensitivity profiles, as well as to more physiologically relevant 3D or in vivo models. In addition, the use of functionalized nanoparticle formulations and more controlled targeting strategies would be of interest to better approximate clinically relevant conditions and to evaluate how surface chemistry and uptake mechanisms influence the observed effects.

Finally, the developments at the CNA cyclotron external beamline including the optimization of the experimental setup, the implementation of a new calibration procedure using Mylar foils to preserve liquid in the biological samples for rapid coverage, and the deployment of a robotized irradiation and positioning system provides a robust platform to further investigate these phenomena. Notably, such experimental capabilities are not widely available, as only a limited number of particle accelerators and research centres are suitably adapted to support this type of radiobiological experimentation. These advancements will enable more controlled and reproducible radiobiology studies in the future.

## 6 CONCLUSION

This study demonstrates that spherical, unfunctionalized 50 and 20 nm AuNPs are non‐toxic to HeLa cells for incubation times up to 24 h, they are efficiently internalized, predominantly accumulating within the cytoplasm and do not produce ROS.

Radiobiology experiments using 12.6 MeV proton irradiations on HeLa cells, with and without AuNPs, quantitatively confirmed the radiosensitizing effect in the presence of AuNPs at doses from 4 Gy, with the enhancement factor increasing progressively with radiation dose. Two independent experimental campaigns yielded consistent SER values of 1.53 ± 0.07 in 2022 and 1.3 ± 0.2 in 2023 with 50 nm diameter gold nanoparticles. Experiments in 2023 using 20 nm AuNPs yielded a similar SER of 1.38 ± 0.18, indicating no significant size‐dependent effect under the tested conditions. Survival curves fitted to the LQ model revealed a systematic increase in the α parameter in the presence of AuNPs. In addition, AuNPs can amplify the ROS production when an oxidative stimulus is present in the cells. The observed increase of DNA damage in the presence of AuNPs, is in line with the observed reduction in clonogenic survival.

Overall, these findings confirm and quantify the radiosensitizing effect of AuNPs under proton irradiation on Hela cells and validates the experimental methodology, providing a solid basis for future investigations into the mechanisms underlying the observed radiosensitization effect.

## FUNDING

This work was supported by the Generalitat Valenciana through the grant CDEIGENT2021/012 and CIPROM/2022/062. Also by the Spanish Ministry of Science and Innovation (MCIN/AEI/FEDER/UE) through grants PID2021‐123879OB‐C21, PID2022‐140603NB‐100 and PID2023‐152568NB‐100, funded by MCIN/AEI/10.13039/501100011033/ and by “ERDF A way of making Europe” as well as by the Ideas Semilla Project IDEAS222767JIME from the Spanish Association Against Cancer (AECC). Furthermore, financial support was provided by the European Union–NextGenerationEU and the Regional Ministry of University, Research and Innovation of the Junta de Andalucía through the Recovery, Transformation and Resilience Plan (PRTR) and the Complementary Plan in Astrophysics (subproject C17.I01.P01.S17, public grant ASTRO21/1.4/4).

## CONFLICTS OF INTEREST STATEMENT

The authors declare no conflicts of interest.

## Data Availability

All data generated and analysed during this study are available from the corresponding authors upon reasonable request.
